# Epigenetic Mechanisms in Bone Biology and Osteoporosis: Can They Drive Therapeutic Choices?

**DOI:** 10.3390/ijms17081329

**Published:** 2016-08-12

**Authors:** Francesca Marini, Luisella Cianferotti, Maria Luisa Brandi

**Affiliations:** Department of Surgery and Translational Medicine, University of Florence and Metabolic Bone Diseases Unit, University Hospital of Florence, Largo Palagi 1, 50139 Florence, Italy; f.marini@dmi.unifi.it (F.M.); luisella.cianferotti@unifi.it (L.C.)

**Keywords:** gene expression, histone modifications, DNA methylation, microRNAs, precision medicine, fragility fracture

## Abstract

Osteoporosis is a complex multifactorial disorder of the skeleton. Genetic factors are important in determining peak bone mass and structure, as well as the predisposition to bone deterioration and fragility fractures. Nonetheless, genetic factors alone are not sufficient to explain osteoporosis development and fragility fracture occurrence. Indeed, epigenetic factors, representing a link between individual genetic aspects and environmental influences, are also strongly suspected to be involved in bone biology and osteoporosis. Recently, alterations in epigenetic mechanisms and their activity have been associated with aging. Also, bone metabolism has been demonstrated to be under the control of epigenetic mechanisms. Runt-related transcription factor 2 (RUNX2), the master transcription factor of osteoblast differentiation, has been shown to be regulated by histone deacetylases and microRNAs (miRNAs). Some miRNAs were also proven to have key roles in the regulation of Wnt signalling in osteoblastogenesis, and to be important for the positive or negative regulation of both osteoblast and osteoclast differentiation. Exogenous and environmental stimuli, influencing the functionality of epigenetic mechanisms involved in the regulation of bone metabolism, may contribute to the development of osteoporosis and other bone disorders, in synergy with genetic determinants. The progressive understanding of roles of epigenetic mechanisms in normal bone metabolism and in multifactorial bone disorders will be very helpful for a better comprehension of disease pathogenesis and translation of this information into clinical practice. A deep understanding of these mechanisms could help in the future tailoring of proper individual treatments, according to precision medicine’s principles.

## 1. Introduction

Osteoporosis, a metabolic skeletal disorder, is a consequence of disrupted normal bone turnover, resulting in reduction of bone mass and mineralization and severe alteration of bone micro-architecture. As a consequence of these structural modifications, osteoporotic bone tissue presents reduced strength that is associated with a high risk of fragility fractures. It is a complex multifactorial disorder resulting from an interaction between genetic and environmental factors, dietary habits, and lifestyle. The focal point of osteoporosis is the deregulation of bone turnover. Normally, in adult skeleton bone remodelling is maintained in a tight balance by highly regulated differentiation, activity, and apoptosis of bone-forming osteoblasts and bone-resorbing osteoclasts. Differentiation of osteoblasts and osteoclasts is finely tuned by profound changes in the expression of numerous regulatory genes. Changes in the expression of these genes could result in altered bone homeostasis and the manifestation of osteoporosis. Family and twin studies confirmed the importance of heritability and genetic profiles in the determination of bone phenotypes, bone mineral density (BMD), and osteoporosis risk. Numerous genes have been related to the regulation of bone metabolism, and some of their polymorphisms have been associated with variations in bone mass, osteoporosis predisposition, and fracture risk. However, together these genetic variants account for less than 10% of the phenotypic variance in BMD [1].

**Figure 1:**
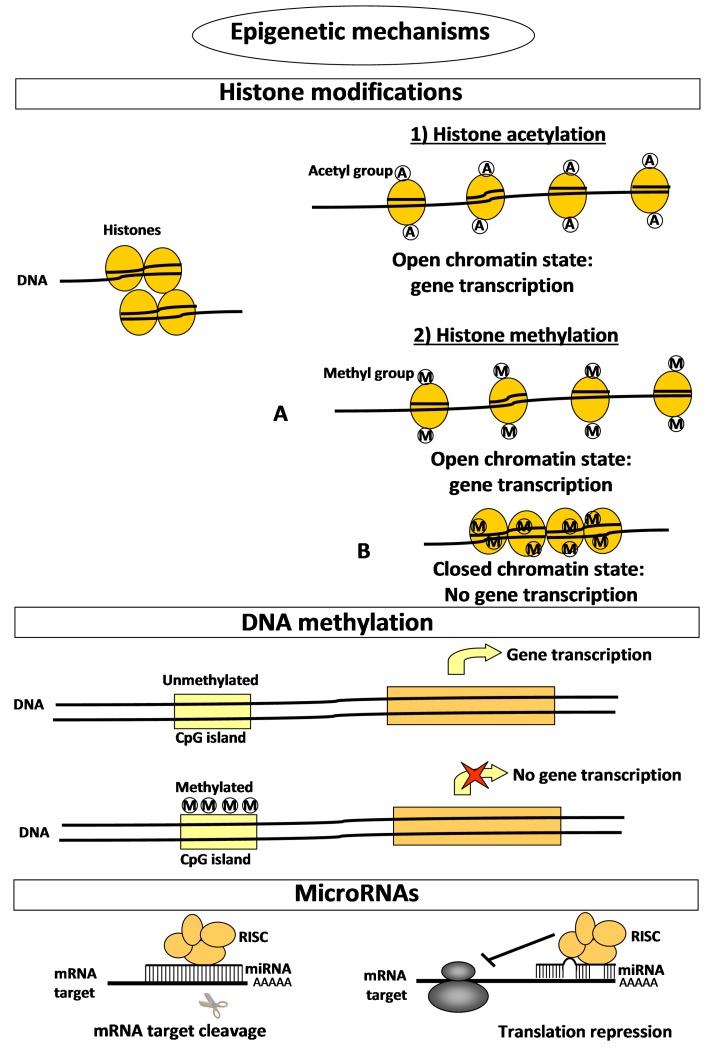
Schematic representation of the mechanism of action of epigenetic mechanisms. Histone acetylation positively regulates gene expression by inducing the opening of chromatin conformation and, thus, favouring the binding of transcription machinery. Histone methylation promotes the opening (panel **A**) or closing (panel **B**) of the chromatin conformation depending not only on the specific lysine residue modified, but also on its degree of methylation (Table 1). In this way, histone methylation can specifically induce or repress gene expression. MicroRNAs (miRNAs) suppress gene expression by selectively binding to the 3′ non coding region (3′UTR) of their mRNA targets through base-pairing. miRNAs can negatively regulate gene expression by two different post-transcriptional mechanisms: the cleavage of the mRNA target or the physical blocking of translation machinery. The choice of mechanism of action is determined only by the nucleotide complementarity between the miRNA and its mRNA target: the miRNA will cleave the target when it has sufficient complementarity to the miRNA itself, or it will repress translation, by physically blocking ribosome activity, if the mRNA does not have sufficient complementarity. In the first case, after the cleavage the miRNA remains intact and active and can proceed to the cleavage of other mRNA targets.

Given the dynamic nature of bone functions, such as to be a major reservoir of essential ions such as calcium and phosphate, this tissue has to provide very rapid responses to organic changes and requirements, which are constantly granted by numerous and complex epigenetic regulatory mechanisms of gene expression. Epigenetics consists of modular changes in gene and protein expression that are independent of inherited DNA nucleotide sequences, and include histone modifications, DNA methylation, and post-transcriptional microRNA (miRNA)-mediated negative regulation of target mRNAs (Table 1 and Figure 1).

**Figure 2:**
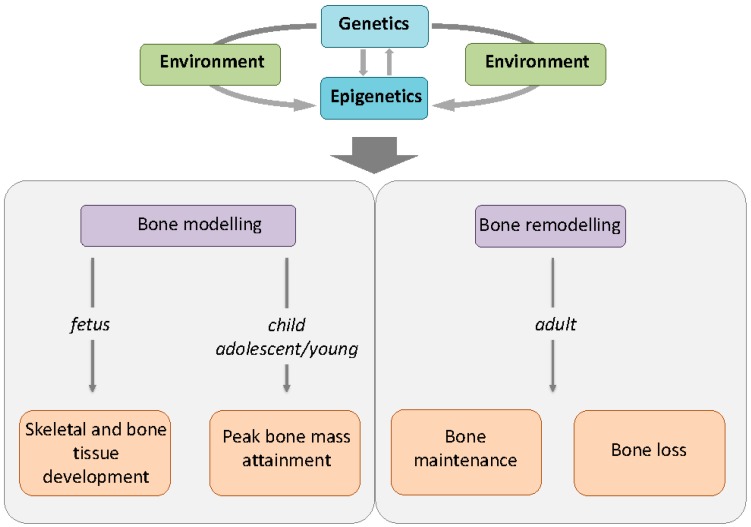
Schematic representation of the role of genetics and epigenetics in bone development and maintenance. Static genetic traits and dynamic epigenetic marks interact with inner and outer environmental stimuli to determine bone features at all ages. While genetics may modulate the expression of epigenetics marks, epigenetic markers can regulate the expression of many genes coding for key molecules driving skeletal modelling in growing bone and remodelling in adult bone. Thus, all processes from bone development to peak bone mass attainment and maintenance can be influenced by epigenetic signatures, implying the possibility of modulating epigenetics in order to prevent/treat bone deterioration.

Epigenetic modifications are all reversible, highly dynamic, age-, cell-, and tissue-specific, and sensitive to endogenous signals and/or environmental stimulation. Every individual may have multiple epigenomes that can rapidly change during development, lifetime, and aging, in response to internal and external signals, both in physiology and physiopathology. Epigenetic processes are currently considered key mechanisms by which environmental factors and stochastic endogenous and exogenous stresses may promote the development of numerous complex multifactorial pathologies, such as osteoporosis. Moreover, recently, modification of epigenetic mechanisms and their activity has also been associated with ageing. In this light, epigenetics could represent a link between the genome and the environment, strongly influencing bone phenotypes and osteoporosis risk, together (and also synergically) with inherited genetic variants, which could explain why genetic factors alone are not sufficient to explain the predisposition to osteoporosis development and fracture occurrence (Figure 2).

Alterations in one or more epigenetic mechanisms can be associated with deregulation of bone homeostasis, as described in detail in the next sections, and subsequent changes of bone-related gene expression may help to account for the “missing heritability” of osteoporosis risk. Moreover, the study and knowledge of these mechanisms, in normal or pathological bone tissues, may help both in assessing osteoporosis and fracture risk and in driving therapeutic options, as well as in identifying novel therapeutic anti-fracture molecules. Establishing the epigenetic signature in an individual could help in tailoring appropriate treatments, according to the science of precision medicine.

Herein, the main epigenetic mechanisms involved in skeletal physiology and pathophysiology will be described.

## 2. Post-Translational Histone Modifications in Bone Biology

**Figure 3:**
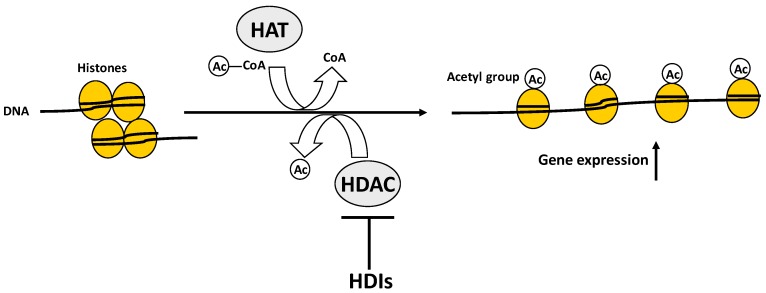
Schematic representation of the role of histone acetyltransferases (HATs) and histone deacetylases (HDACs). HATs induce histone acetylation by transferring an acetyl group from the acetyl coenzyme A to histone lysine side chains (mostly at histone 3 lysine 4 (H3K4Ac) or histone 3 lysine 9 (H3K9Ac)), inducing the opening of chromatin status and promoting gene transcription. Conversely, HDACs remove the acetyl groups from histones, inducing the closing of chromatin status and blocking gene transcription. Histone deacetylase inhibitors (HDIs) inhibit the catalytic activity of HDACs by directly binding to their catalytic sites.

Post-translational histone modifications include a large number of histone changes, such as methylation, acetylation, phosphorylation, ubiquitination, ADP ribosylation, sumoylation, deamination, and non-covalent proline isomerisation [2]. These modifications, induced or removed by specific enzymes, alter the structure of chromatin, exposing or hiding gene promoters and, thus, respectively, promote or repress gene transcription, through a dynamic phenomenon called the “histone code”. One of the principal histone modifications is the acetylation/deacetylation process that adds or removes acetyl groups to histone lysine side chains. Histone acetylation usually relaxes the chromatin, allows transcription factors and RNA polymerase II to access the DNA, and, thus, is associated with the promotion of gene transcription, while histone deacetylation induces compaction of the chromatin and is responsible for blocking gene expression. Histone acetyltransferases (HATs) and histone deacetylases (HDACs) act in a counteractive manner to regulate histone acetylation status in response to incoming signals, and are involved in the control of numerous important biological processes (Figure 3).

Bone remodelling has also been demonstrated to be sensitive, at various cellular levels, to histone modifications. It is conceivable that these enzymes could rapidly regulate bone turnover in response to endogenous or external changes. Eighteen HDAC enzymes have been identified in humans, and several of them have been demonstrated to strongly contribute to regulate the correct skeletal development and accrual, bone mass peak acquisition, and optimal bone mass maintenance during lifetime and aging. These enzymes regulate histone acetylation status and directly deacetylate (i.e., inactivate) key proteins that drive bone homeostasis, such as the early osteoblast differentiation regulator Runt-related transcription factor 2 (RUNX2) (Table 2). HDACs are divided into four classes (I, II, III, and IV) based on their structure, function, cellular localization, and expression. Classes I, II, and IV (commonly referred as HDACs) present a zinc-dependent catalytic sites, while class III (commonly named as Sirtuins; Sirts) needs NAD^+^ for the catalytic action.

HDAC1 and HDAC2 are highly homologous and can form hetero- or homo-dimers with each other. They both play a role in suppressing osteoblastogenesis, and levels of their mRNAs are usually decreased during osteoblast differentiation to grant the correct development of this process [3]. Recently, it has been shown that HDAC2 expression is increased during receptor activator of nuclear factor-κB ligand (RANKL)-induced osteoclast differentiation of bone marrow-derived precursors [4]. It was revealed to be a key regulator of osteoclastogenesis by activating Akt kinase that directly phosphorylates and inhibits Forkhead box protein O1 (FoxO1), a negative regulator of osteoclast differentiation [4].

HDAC3 is highly expressed in osteoblasts at all stages of differentiation, and it directly interacts with the transcription factor RUNX2 [5]. This enzyme was demonstrated to be important for bone mass acquisition and maintenance during aging, and for correct craniofacial development. Mouse models deficient in HDAC3, in osteochondral precursor cells, showed reduced bone length and decreased cortical and trabecular bone mass and mineralization, severe osteoporosis, and suffered frequent fragility fractures [6]. The complete germline knockout of *HDAC3* is lethal at the embryonic level [7]. Conversely, the RNA interference-driven in vitro suppression of HDAC3 in pre-osteoblasts increases matrix mineralization [5].

HDAC4 has been shown to directly deacetylase RUNX2, repressing its transcriptional activity and promoting its degradation in mature osteoblasts [8]. Loss of HDAC4 in mouse models accelerates endochondral bone formation, inducing premature ossification of multiple cartilaginous sites that lead to severe skeletal defects, including shortened long bones, vertebral body fusion, and premature endochondral ossification of the skull. Conversely, the over-expression of Hdca4 results in a severe deficit of endochondral ossification and slows the ossification of cartilage in vivo [9]. Intramembranous bone formation is normal in both cases, proving that HDAC4 has a role specifically in regulating the process of endochondral ossification from cartilage. HDAC4 also directly controls bone morphogenetic protein (BMPs) and TGFβ signalling during bone development [10]. In humans, loss-of-function mutations of the *HDAC4* gene were found to be the cause of Brachydactyly-Mental Retardation syndrome, characterised by distinctive craniofacial features and shortened metacarpal and metatarsal bones.

Conversely, HDAC8 has a crucial role in intramembranous ossification. Indeed, germline deletion of this gene in mice is detrimental to skull bone formation and leads to perinatal death [11]. In humans, inactivating mutations of the *HDAC8* gene has been identified in patients with Cornelia de Lange syndrome and with a clinical condition similar to Wilson-Turner linked mental retardation syndrome, both characterized by shortened long bones and distinctive craniofacial dimorphisms [12,13], the former also by delayed fontanel closure and small hands and feet.

HDAC5 is expressed in mature osteoblasts where, in association with HDAC4 and TGFβ1, it represses the transcriptional activity of RUNX2 [8], and also directly deacetylates RUNX2, promoting its Smurf-mediated degradation. Two related young patients with primary juvenile osteoporosis showed elevated HDAC5 protein level and reduced RUNX2 expression in bone [14].

HDAC7 is highly expressed both in mature osteoblasts and in osteoblast precursors, and it has been demonstrated to repress RUNX2 by deacetylation-independent mechanisms [15] and to be necessary for endochondral ossification. In vitro suppression of HDAC7 by RNA interference favours osteoblast differentiation in a bone morphogenetic protein 2 (BMP2)-dependent manner [15]. Inactivation or suppression of HDAC7 expression results in proliferation of osteoclast precursors and promotion of RANKL-induced osteoclastogenesis [16]. Also, *HDAC9* knock-out mice showed an increased number of active osteoclasts, a higher bone resorption, reduced bone formation indices, and an osteopenic phenotype. Conversely HDAC9 expression suppresses osteoclastogenesis, presumably by negatively regulating Rankl expression. These data suggest a possible involvement of HDAC7 and HDAC9 in osteoporosis [17].

Sirt1 and Sirt6 are the only class III HDACs that have shown, to date, a proven role in bone tissue development and metabolism [17]. Activation of Sirt1 in mesenchymal stem cells (MSCs) promotes osteogenic differentiation, blocking adipocyte differentiation. Conversely, inhibition of Sirt1 expression results in a promotion of adipogenic differentiation [18]. Ovariectomy and subsequent estrogen depletion result in Sirt1 protein absence in animal models; this could explain the increased adiposity in bone marrow and the rapid bone loss after menopause. The restoration of Sirt1 expression could reveal a possible approach to preventing post-menopausal osteoporosis. Both Sirt1 and Sirt6 facilitate endochondral ossification—Sirt6, particularly, by controlling chondrocyte proliferation and differentiation.

Histone deacetylase inhibitors (HDIs, i.e., valproic acid, trichostatin A) are small molecules able to inactivate HDACs (Figure 3) by binding to their zinc-containing catalytic sites. HDIs have been demonstrated to induce a transient increase in osteoblast proliferation and viability, to enhance osteoblast differentiation in vitro, to augment alkaline phosphatase (ALP) production and expression of type I collagen, osteopontin (OPN), bone sialoprotein, osteocalcin (OCN), and RUNX2, and to accelerate the extracellular mineralization in osteogenic cell lines and mesenchymal progenitor cells [19]. Moreover, these molecules induce MSCs to differentiate into mature osteoblasts. HDIs have also been demonstrated to block the formation of pre-osteoclast-like cells and their fusion into multinucleated osteoclast-like cells in rat bone marrow cell culture, and to reduce osteoclast-specific synthesis of cathepsin K [20], and to act on mature osteoclasts, causing their apoptosis [21]. The increased acetylation of histones H3 and H4, at the RANKL promoter, favours RANKL transcription, indicating that epigenetic chromatin remodelling is involved in RANKL expression [22] and, thus, in the regulation of osteoclast activation.

These in vitro results suggest a possible application of HDIs as anti-fracture therapy in osteoporosis, able both to reduce osteoclast bone resorption and promote osteoblast bone formation. However, both in vivo studies in animal models [23,24] and human epidemiological studies on HDI treatment for epilepsy and mental disorders [25,26] evidenced a negative effect of prolonged HDI therapy on BMD value, associated, in some cases, with a higher risk of fracture [27]. The molecular causes of controversial results between HDI effects on bone in vitro or in vivo, as well as the exact mechanisms by which HDIs reduce BMD in vivo, remain to be fully elucidated. Current HDIs act on various different HDACs, which play crucial roles at various points of osteoblastogenesis, so that long-term HDI therapy can affect all of them at the same time. Ideal HDIs should target only one specific HDAC. With this in mind, a new generation of drugs of this kind is currently under development.

Recently, a study by Feng et al. tested the ability of resveratrol, a plant polyphenol acting as an agonist of Sirt1, to promote osteogenesis in vitro and prevent bone loss in animal models and, thus, its possible protective effect on osteoporosis [28]. In rats with bilateral ovariectomy, resveratrol moderately restored the serum level of ALP and OCN and improved bone structure. Moreover, resveratrol promoted the in vitro osteoblast differentiation of bone marrow mesenchymal stromal cells via the Sirt1-NF-κB signalling pathway. The authors demonstrated that the beneficial bone effects of resveratrol were exerted by its direct action on Sirt1, since the RNA silencing of *Sirt1* resulted in a loss of any positive effect of resveratrol administration. These results suggested a therapeutic potential of resveratrol against post-menopausal osteoporosis.

Histone methylation is another common histone modification, finely tuned by the opposing enzymatic actions of histone lysine methyltransferases (KMTs) and histone lysine demethylases (KDMs). The histone methylation state has been revealed to be an important modulator of MSC differentiation into the osteogenic lineage or the adipogenic lineage. SETDB1 and EZH2 are two KMTs that inhibit osteogenic differentiation of MSCs via blocking *RUNX2*, transcription factor 7 (*TCF7*), and Osterix (*OSX*) transcription, and promote adipogenesis of MSCs by inducing the expression of peroxisome proliferator-activated receptors gamma (*PPARγ*), myocyte enhancer factor 2 interacting transcriptional repressor (*MITR*), and Wnt-related genes [29]. Conversely, KDM4B, KDM6A, and KDM6B are KMTs that promote osteogenesis and repress adipogenesis by inducing the transcription of *RUNX2*, *BMP*, *OCN*, distal-less homeobox 5 (*DLX5*), and HOX-related genes [29]. Other numerous KMTs are involved in the alternative regulation of these two differentiation processes and, thus, in normal bone turn-over or pathologies, and they have been extensively reviewed elsewhere recently [29].

## 3. DNA Methylation in Bone Biology

DNA methylation consists of the reversible covalent addition of a methyl group to the fifth carbon of a cytosine residue (5-mC) located in the CpG islands of gene promoters, and it is commonly associated with the repression of gene expression. The enzymes responsible for DNA methylation are de novo DNA methyltransferases (DNMT3A and DNMT3B) that act preferentially on unmethylated and hemimethylated DNA and are important during embryo development, and maintenance methyltransferase (DNMT1) with a preference for hemimethylated DNA and able to maintain the methylation patterns during DNA replication. Demethylation is both a passive reduction of methylation during DNA duplication and an active mechanism not yet fully elucidated.

DNA methylation state has an important role in both bone metabolism and age-related disorders, such as osteoporosis. Methylation of several genes is involved in the regulation of bone cell differentiation and in the normal bone remodelling process. In osteoblasts, DNA methylation state co-regulates the expression of various important genes involved in bone cell functions, such as alkaline phosphatase (*ALPL*), sclerostin (*SOST*), *OSX*, *DLX5*, oestrogen receptor alpha (*ESR1*), *OPN*, *RANKL*, osteoprotegerin (*OPG*), secreted frizzled-related protein 1 (*SFRP1*), and leptin (*LEP*) [30]. *ALPL* promoter methylation is inversely associated with gene transcription and expression and is differently regulated at different stages of osteoblast differentiation. Indeed, osteoblasts show poor *ALPL* methylation, bone lining cells have an intermediate methylation status, and an *ALPL* hypermethylation is characteristic of osteocytes, associated with no expression of *ALPL* gene. In parallel, transition from osteoblasts to inactive osteocytes is associated with a progressive reduction in levels of *SOST* promoter methylation.

Little is known about DNA methylation and the regulation of osteoclast differentiation and activity. It can be also inferred that DNA methylation has a direct role in the pathogenesis of osteoporosis, but, to date, few studies directly support this hypothesis, and results are still inconclusive.

Recently, a study by Nishikawa et al. [31] demonstrated that Dnmt3a positively regulates osteoclast differentiation by methylation, and expression induction, of the S-adenosylmethionine (*SAM*) gene. SAM protein expression results in the induction of osteoclastogenesis by repression of anti-osteoclastic genes, such as *RANKL*. The authors also showed that mice osteoclast precursors deficient in *Dnmt3a* were unable to efficiently differentiate into mature osteoclasts in vitro, and that *Dmnt3a* knock-out mice model showed a reduced number of active osteoclasts and a higher bone mass with respect to the normal control. Furthermore, they investigated the possibility that targeted Dnmt3a inhibition may have a positive effect in preventing bone loss, by administration of the Dnmt3a inhibitor theaflavin-3,3′-digallate (TF-3) to ovariectomized female mice, as a model of postmenopausal osteoporosis. Treatment with TF-3 resulted in a reduction of number of activated osteoclasts, a higher bone mass, and protection against bone loss, suggesting that the inhibition of Dnmt3a could be a beneficial strategy for preventing bone loss and controlling postmenopausal osteoporosis. Human clinical trials on osteoporosis and other bone disorders characterized by excessive osteoclast activity are surely needed to confirm this preliminary result and to design a possible novel therapeutic approach for these diseases.

Analysis of DNA methylation in bone tissue samples from osteoporotic subjects, with respect to control individuals, revealed a hypermethylation of *OPG* and *RANKL* CpG islands, which influences the transcription level of these genes and, subsequently, the osteoclastogenesis [32]. However, the concomitant increase of the RANKL: OPG ratio in patients with osteoporotic fractures seemed to be derived from other methylation-independent mechanisms [32].

A case-control study analysed DNA methylation at four regions upstream of the *SOST* transcription start site in bone biopsies from post-menopausal osteoporotic women and normal BMD controls, indicating a statistical difference in methylation state between affected patients and the healthy group [33]. The authors suggested that the increased methylation of *SOST* promoter in osteoporosis could be a mechanism that, through the reduction of serum SOST protein level and, subsequently, the enhancement of canonical Wnt signalling, aims to induce compensatory bone formation, counteracting osteoporosis-associated bone loss.

A genome-wide study evaluated the DNA methylation profile, at the genome level (methylation of 23,367 GpG sites in 13,463 genes was analysed), in femoral head trabecular bone specimens from osteoporotic hip fractures with respect to hip samples of patients with osteoarthritis [34]. The results showed an inverse correlation between methylation and whole gene expression in both cases. A significant difference in methylation level was found in 241 CpG sites located in 228 genes, the great majority of them involved in glycoprotein, neuronal differentiation, adherence, homeobox, and cell proliferation pathways. Among these, the homeobox superfamily genes, differentially methylated between osteoporosis and osteoarthritis, include genes that are directly involved in skeletal embryogenesis. It could be hypothesised that the existence of one or more developmental regulatory components, probably including DNA methylation, acting during development and growth, is responsible for correct skeletal formation and homeostasis and also for the further development of osteoporosis or osteoarthritis during aging.

Environmental factors have been associated with DNA methylation and skeletal development during the early phase of embryogenesis in animal studies and in humans. Indeed, maternal dietary habits influence the bone mass of the progeny [35,36], presumably also through the induction of methylation variations and/or changes in histone modifications in genes important for bone cell differentiation and function, such as *PPARγ* and glucocorticoid genes [37,38].

DNA methylation patterns are highly cell-specific, and can differ between different stages during a lifetime or in correlation with diseases. Therefore, they could be used as viable diagnostic, prognostic, and/or therapy-guiding biomarkers in numerous biological samples, thanks also to the great stability of methylation in spite of external perturbations and sample manipulations. In this light, large-scale studies comparing methylation states between cases and relative controls can identify this epigenetic trait associated with specific disorders. Nevertheless, unlike in tumours, where DNA methylation screenings have validated clinical applications, no methylation tests are currently available or under investigation for common bone disorders.

## 4. miRNAs in Bone Biology

miRNAs are endogenous single-stranded small non-coding RNAs that negatively regulate gene expression, at the post-transcriptional level, by selectively binding to the 3′ non-coding region (3′UTR) of specific target mRNAs through base pairing. By this mechanism, miRNAs can suppress the expression of their target proteins by directly blocking the translation process or enhancing the degradation of mRNAs. Over 2000 human miRNAs have been identified, to date, all of them involved in the regulation of important biological processes such as development, cell growth, and cell differentiation. The fundamental role of miRNAs in the regulation of growth and differentiation of both osteoclasts and osteoblasts has been clearly assessed. Indeed, the genetic deletion of Dicer, the enzyme responsible for maturation and activation of all miRNAs, in osteoblast progenitors [39] and pre-osteoclast lineage cells [40] resulted, respectively, in severe impairment of osteoblast differentiation and maturation with subsequent alterations in bone tissue structure and matrix mineralization, or in a reduced number of active osteoclasts with subsequent decreased bone resorption and osteopetrosis. Dicer excision in differentiated osteoblasts results in an increased bone mass in adult mice, with double cortical bone width and trabecular thickness with respect to normal controls [39].

In recent years, numerous miRNAs have been demonstrated to directly regulate, either positively or negatively, osteoblastogenesis and osteoclastogenesis, by interacting with specific factors involved in the control of these two processes. Herein, a brief overview of the principal miRNAs involved in the regulation of osteoblast or osteoclast differentiation is given, with the description of their specific action in these processes.

Fourteen miRNAs (miR-23a, miR-30a-d, miR-34c, miR-133a, miR-135a, miR-137, miR-204, miR-205, miR-211, miR-217, miR-218, miR-335, miR-338, miR-433, and miR-3077-5p) target the 3′UTR of *RUNX2*, the master transcription factor in osteoblast differentiation [41,42,43,44]. All these miRNAs control osteogenic differentiation at different levels, blocking the osteoblast lineage progression of committed pre-osteoblasts or directing multi-potent mesenchymal stem cells toward adipogenic differentiation. Conversely, two miRNAs, miR-2861 and miR-3960, have been shown to indirectly induce *RUNX2* expression by targeting its inhibitors *HDAC5* and *HOXA2*, and, thus, promoting osteoblast differentiation [45]. miR-2861 and miR-3960 are transcribed together from the same miRNA polycistron during BMP2-induced osteogenesis. Over-xpression of miR-2861 and miR-3960 promotes BMP2-induced osteoblastogenesis, while their inhibition reduces osteoblastogenesis. RUNX2 itself was demonstrated to directly bind to the promoter of miR-3960 and miR-2861, positively regulating their expression. Moreover, it has been shown that RUNX2 negatively regulates the expression of the miR cluster 23a-27a-24-2, by DNA direct binding to specific elements in the promoter of these genes [46]. The reduced expression of this miR cluster is responsible for the promotion of osteoblast differentiation. Taken together, all these data suggest that the RUNX2 factor establishes complex regulatory networks with miRNAs that play, in this way, a central role in the control of correct progression and maintenance of the osteoblast phenotype.

A homozygote mutation of the gene encoding miR-2861 has been associated with a rare form of primary juvenile osteoporosis in two adolescents [14]. Consistent with data obtained from mouse models, bone samples from these two patients showed increased levels of HDAC5 and decreased levels of RUNX2, confirming that miR-2861 is an important physiological regulator of osteoblast differentiation. Thus, deregulation of miR-2861 can contribute to the establishment of osteoporosis via its effect on osteoblasts and could be an optimal target for future anti-osteoporotic therapies.

Also, BMPs and the Wnt signalling pathway, fundamental and key mechanisms driving skeletal-related gene expression involved in the formation of cartilage and bone, have been demonstrated to interact with various miRNAs to control osteoblast differentiation.

BMP2 controls the switch between muscle and bone differentiation by regulating miRNA expression. BMP2 has been shown to downregulate 22 miRNAs that inhibit the translation of crucial factors of osteoblastogenesis, by directly targeting their 3′UTR, such as *SMAD5* (the intracellular receptor of BMP2 within pre-osteoblasts) and *RUNX2* [42]. Indeed, two miRNAs downregulated by BMP2, miR-135 and miR-133, are, respectively, negative regulators of *SMAD5* and *RUNX2*. miR-135 inhibits directly *SMAD5* expression, and, thus, indirectly also *RUNX2* expression. miR-133 showed the dual function of promoting myogenesis and, at the same time, inhibiting differentiation of MSCs into the osteoblast lineage.

Specific miRNAs were shown to regulate the Wnt/β-catenin signalling pathway, interfering with osteoblast differentiation. miR-29a gene expression is enhanced by canonical Wnt signalling, and its expression is increased during osteogenic differentiation of mesenchymal osteoblast precursors [47]. miR-29a targets and inhibits expression of Dikkopf-1 (DKK1), Kremen2, and secreted frizzled related protein 2 (SFRP2), three key negative regulators of Wnt signalling, thus potentiating the effect of Wnt signalling, promoting the expression of Wnt-regulated genes, and favouring osteoblast differentiation and bone formation.

Wnt signalling also induces the expression of miR-128 during osteoblast differentiation, while miR-128 promotes osteogenic commitment and differentiation of bone marrow MSCs by directly inhibiting the translation of three repressors of Wnt signalling, SOST, Dickkopf-2 (DKK2), and SFRP2, through a positive feedback loop [48].

Additional miRNAs enhance osteoblast differentiation through the modulation of the canonical Wnt pathway. miR-335-5p activates Wnt signalling and promotes osteogenic differentiation by downregulating DKK1, and is highly expressed in both osteoblasts and hypertrophic chondrocytes during embryogenesis [49]. Treatment of pre-osteoblasts with anti-miR-335-5p reverses the effect of miR-335-5p and reduces osteoblastogenesis. miR-27 is overexpressed during osteoblast differentiation, and its inhibition is associated with blocking of cell differentiation [50]. miR-27 directly targets and inhibits adenomatous polyposis coli (*APC*) gene expression, a fundamental component of the axin complex that positively regulates phosphorylation and subsequent degradation of β-catenin, by blocking the Wnt signalling. Inactivation of *APC* by miR-27 induces the accumulation of β-catenin, its translocation into the nucleus, and the transcription of Wnt-regulated genes, resulting in a promotion of osteogenesis. Increased expression of miR‑142‑3p has been seen during osteoblast differentiation of mesenchymal precursors [51]. This miRNA acts in the same way as miR-27, by targeting and repressing *APC*.

**Figure 4:**
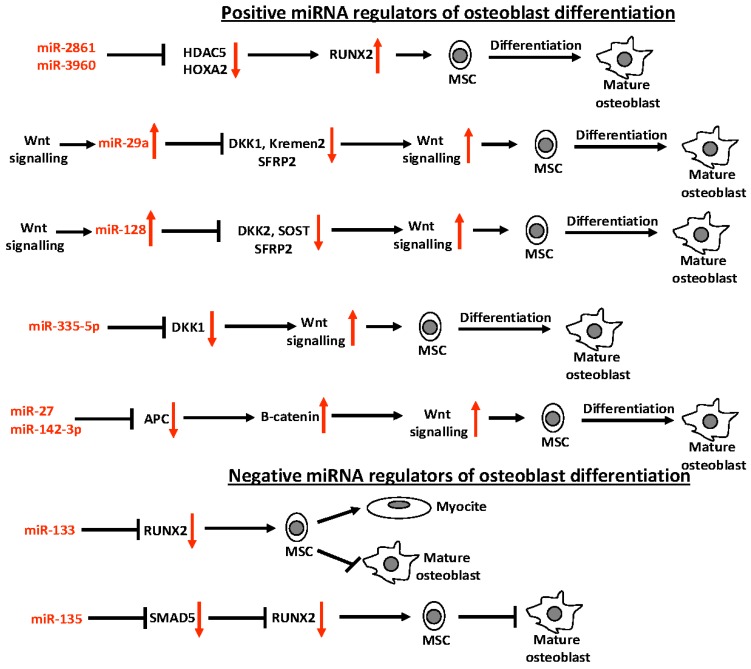
Schematic representation of positive (↑) and negative (↓) miRNA regulators of osteoblast differentiation.

All miRNAs described above are important positive or negative mediators of osteoblast differentiation (Figure 4), and could be novel promising targets for the development of preventive or therapeutic agents against osteogenic disorders.

**Figure 5:**
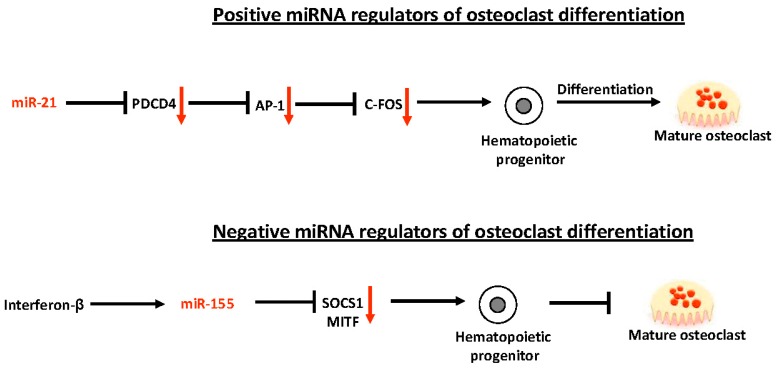
Schematic representation of positive (↑) and negative (↓) miRNA regulators of osteoclast differentiation.

The involvement of miRNAs in the regulation of osteoclast differentiation has been markedly less investigated (Figure 5).

Three principal miRNAs have been associated with the regulation of osteoclastogenesis: miR-21, miR-155, and miR-223. A significant stimulation of miR-21 during RANKL-induced osteoclastogenesis has been found [52]. miR-21 targets programmed cell death 4 (*PDCD4*). The PDCD4 protein is known to directly negatively affect the activity of the transcription factor AP1. This results in decreased expression of AP1-regulated genes such as *c-FOS*, an important transcription factor for inducing osteoclast differentiation and osteoclast-specific downstream target genes. Diminished expression of PDCD4, by miR-21, removes the repression from c-FOS expression and promotes osteoclast differentiation. Moreover, it has been shown that RANKL-induces c-FOS expression that upregulates miR-21 expression in a positive feedback loop responsible for promotion of RANKL-mediated osteoclastogenesis. The high expression of miR-21 promoting osteoclast differentiation is inhibited by oestrogens [53]. In particular, oestrogens downregulate miR-21 biogenesis, resulting in an increase of Fas Ligand (FasL, a target of miR-21), which induces the apoptosis of osteoclasts [53]. These data could suggest miR-21 as a target for novel antagomir-based antiresorptive therapy as an alternative to oestrogen replacement therapy in post-menopausal women.

Conversely, miR-155 has a suppressive effect on osteoclast differentiation in vitro, mediated by its inhibition of expression of *SOCS1* and *MITF*, two essential positive regulators of osteoclastogenesis [54]. Expression of miR-155 is induced by interferon-β, which, in this way, exerts negative action on osteoclast differentiation. miR-155 could represent a novel viable agent for the treatment of osteoclast-mediated diseases, such as osteoporosis, but its systemic role in osteoclast suppression and bone remodelling remains to be clearly elucidated.

Another miRNA, miR-223, plays a critical role in osteoclast differentiation. An increased expression of miR-223 blocks the maturation of pre-osteoclastic cells into tartrate-resistant acid phosphatase (TRAP)-positive multinucleated mature osteoclasts, while normal expression of miR-223 favours this cell differentiation [55]. A recent study associated low expression of miR-223 with the promotion of osteoclastogenesis [56]. High concentrations of inorganic phosphate (Pi) decreased miR-223 expression in osteoclast precursors in vitro, resulting in a marked decrease in osteoclastogenesis. miR-223 targets the Nuclear Factor IA (NFIA), a negative regulator of macrophage colony stimulating factor (M-CSF), and Ras homolog gene family ()member B RhoB, a transcription factor involved in cell proliferation. Data from these two studies suggested that both too high or too low a level of miR-223 precludes efficient osteoclast differentiation, and indicated that this miRNA is a promising therapeutic target for bone metabolic disorders characterised by excessive osteoclast activity.

Given their important roles in the normal regulation of osteoblast and osteoclast development and function, deregulation of miRNA activity is surely an important factor in bone disorders. Three mechanisms could be responsible for altered functions of specific miRNAs in bone tissue, leading to the development of osteoporosis or other metabolic bone disorders: (1) mutations in genes encoding miRNAs involved in the regulation of bone metabolism, which deregulate their function and/or their expression; (2) mutations affecting the 3’UTR binding sites of target mRNAs, fundamental for the regulation of transcription, and modulated by miRNAs; (3) abnormalities in miRNA expression altering regulatory networks involved in the control of osteoblast and/or osteoclast differentiation and activity.

Recently, it has been demonstrated that miRNAs are not only active locally within the cells that express them, but can also be secreted via extracellular microvesicles or exosomes. Indeed, circulating miRNAs have been identified in 12 different body fluids [57]. Circulating miRNA signatures have been identified as diagnostic and/or prognostic biomarkers of various disorders, including age-associated diseases. The role of circulating miRNAs as biomarkers for osteoporosis and other metabolic bone disorders has not received much attention until recent years. Indeed, the finding that osteoblasts seem to communicate via exosome shuttling and the discovery that exosome miRNA content changes during osteoblast differentiation both support the possible use of extracellular miRNAs as biomarkers of bone metabolism. Circulating miRNAs have the potential to be surrogate biomarkers (bioproducts) of bone metabolism as well as provide information on the cellular and molecular processes involved in bone turnover and be minimally invasive markers with specific functional relevance to osteoblast, osteoclast, and osteocyte differentiation and activity. Measurement of specific serum miRNAs could help in the near future to identify patients at high risk of osteoporosis and fragility fractures and/or to monitor the efficacy of antiresorptive therapies and bone-forming agents, either used alone or in association with existing biological markers of bone metabolism.

In this setting, few studies have tried to identify circulating miRNAs associated with BMD, osteoporosis, and fracture risk. Li et al. evaluated, by quantitative RT-PCR (RT-qPCR), the expression of three specific miRNAs (miR-21, miR-133a, and miR-146) in the plasma of 120 post-menopausal Chinese women with normal, osteopenic, or osteoporotic range of BMD, evidencing a higher level of miR-133a and a lower level of miR-21 in osteoporotic and osteopenic women with respect to the normal group [58]. Seelinger et al. measured an 83-miRNA RT-qPCR panel in a pool of 10 serum samples from osteoporotic Caucasian men and women vs. a pool from 10 non-osteoporotic Caucasian women, both with hip fractures [59]. Results from PCR-array have been validated on further serum samples from 30 osteoporotic patients vs. 30 normal controls and on miRNA samples isolated from the bone tissue of 20 osteoporotic and 20 non-osteoporotic patients. A signature of nine circulating miRNAs (miR-21, miR-23a, miR-24, miR-93, miR-100, miR-122a, miR-124a, miR-125b, and miR-148a) has been found to be significantly upregulated in patients with osteoporosis, with miR-21, miR-23a, miR-24, miR-25, miR-100, and miR-125b displaying significantly higher expression in bone tissue from osteoporotic patients. Weilner et al. tested a panel of 175 miRNAs on serum samples from seven female patients with recent osteoporotic hip fracture and seven age-matched female controls [60]. Six miRNAs (miR-10a-5p, miR-10b-5p, miR-22-3p, miR-133b, miR-328-3p, and let-7g-5p) showed significantly different serum levels in the presence of a fracture. These data were validated on a further 12 fractured women vs. 11 controls, confirming miR-22-3p, miR-328-3p, and let-7g-5p as differentially expressed in the presence of a fracture. Panach et al. measured a panel of 179 miRNAs in the serum samples of eight osteoporotic women with fractures vs. five controls, and validated data on 15 patients with fractures vs. 12 controls [61]. They determined that miR-122-5p, miR-125b-5p, and miR-21-5p were upregulated in the presence of a fracture. In particular, for miR-21-5p, the difference detected between fractured women and controls was independent of age, and its circulating levels were correlated to those of CTx, a marker of bone resorption.

These studies support a future application of circulating miRNA dosage as a biomarker for assessing osteoporosis status and fracture risk. However, all these studies analysed a small number of samples, strongly reducing the efficacy of association, and they evaluated only restricted panels of miRNAs. Further studies are surely needed, including larger populations and the application of high-throughput technologies, such as next-generation sequencing (NGS), to evaluate, at the same time, all the known human miRNAs, in order to identify specific signatures, possibly driving diagnosis and therapeutic choices.

## 5. Discussion

Epigenetics mechanisms play a fundamental role in regulating biological processes, also in response to exogenous environmental influences. In this light, there is increasing epidemiological and biological evidence that some environmental stimuli, such as viral and bacterial infections, prolonged exposition to chemical agents and pollutants, an imbalance of nutrients, long-term pharmacological treatments, and physical and mental stresses, can induce important epigenetic changes, significantly influencing gene expression and biological processes. They can result in favouring and/or promoting the development of complex multifactorial disease during aging. Since it is now well known that epigenetic factors play a major role in skeletal development and bone maintenance, a deregulation of these regulatory mechanisms, induced by environmental factors (i.e., dietary habits and lifestyle aspects), could be an important determinant of the development of osteoarticular diseases, including osteoporosis, acting in a synergic manner with predisposing genetic determinants. Indeed, bone development at the embryo stage and skeletal growth and bone mass peak acquisition during infancy, adolescence, and early adulthood, as well as bone mass maintenance during aging and bone progressive loss in the elderly, can all be modified by environmental influences such as maternal and perinatal nutrition, dietary habits, physical activity, smoke, alcohol intake, hormonal therapies, etc. The effect of these exogenous factors on bone metabolism is mediated by the epigenetic mechanisms. In particular, nutri-epigenetic studies demonstrated the influence of some nutrients on foetal and/or placental epigenetic mechanisms. Food is more than simple energy for our body and/or the main source of key metabolites for synthesis of biological macromolecules and cell and tissue activity. Nutrients are important determinants of epigenetic functions and can exert variable effects on these endogenous regulatory mechanisms; derived early epigenetic modifications can alter gene expression in a way that may influence long-term health and diseases. Indeed, maternal nutritional imbalance and deficiency not only have a severe effect on correct foetal development but may also have a persistent effect on the health of the offspring and even be transmitted to the next generation [62]. Maternal diet influences post-natal bone mass and size, and birth weight has been positively associated with young adult bone mineral content and bone size [63,64] and with adult hip and spine bone mineral content [65]. Low maternal calcium and vitamin D intake during late pregnancy has been associated with reduced bone mineral content. Maternal under-nutrition or maternal high fat diet were both demonstrated to affect DNA methylation of the foetus during intrauterine development, altering the pre- and post-natal expression of multiple genes that may regulate skeletal growth and bone mass acquisition. Prenatal and perinatal diet-induced DNA methylation changes can persist during life and also for multiple generations. A review by Bocheva et al. [66] gives an interesting overview of the epigenetic modulation of intrauterine and post-natal skeletal development and of placental transfer of nutrients and their future impact on osteoporosis development.

In this light, a better comprehension of the effect of maternal diet on skeletal development and bone mass determination and of the exact underlying epigenetic mechanisms is needed. This will be very helpful to design and activate large-scale public health nutrition programs for pregnant women, infants, and children, in order to reduce the risk of osteoporosis and fragility fracture in the elderly.

Regarding molecules influencing epigenetic mechanisms that could find a future application in the area of treatment of metabolic bone disorders, currently few preliminary data are available on resveratrol, HDIs, and TF-3. The effects of HDIs on bone tissue appear to be controversial and should be better investigated in prospective clinical trials, focusing on the design of novel HDIs not targeting a wide range of HDACs but specific for only one enzyme. Also, the beneficial effect of resveratrol on bone mass and the protective effect of TF-3 on bone loss both need to be confirmed and validated in clinical trials.

Calcium is commonly administered as a supplement for the prevention and treatment of osteoporosis. A recent study [67] showed that in the presence of a high concentration of calcium ions and in the absence of reducing agents, vertebrate DNMTs (principally DNMT1, DNMT3B, and, to a lesser extent, DNMT3A) can act, in vitro, as demethylases and remove the methyl group from the 5-mC, altering the normal DNA methylation status and presumably having an effect on gene expression and the risk of associated disease development. No data are available on the effect of this activity on bone cells, and since the calcium ion concentration (≥10 μm) needed for the in vitro DNA demethylation reaction is higher with respect to the normal intracellular level of calcium, the intervention of other cofactors and/or specific signal transduction pathways could be required for the in vivo demethylation reaction to act.

## 6. Conclusions and Future Perspectives

In conclusion, better comprehension of both normal and deregulated epigenetic processes in bone metabolism will provide insights into normal skeletal development and maintenance, as well as into disease pathogenesis. Increasing advances in high-throughput technologies for epigenetic research, as well as the progressive reduction of the cost-efficiency ratio will enable, in the near future, a better understanding of the epigenetic basis of both normal and pathological bone metabolism, helping us to develop better diagnostic tools, foresee disease development, grant a more favourable prognosis, and identify novel drug target for the design of innovative therapies.

Moreover, since the epigenome—conversely to the genome—is reversible, we could manipulate identified altered epigenetic processes responsible for the development of diseases. Indeed, epigenetic mechanisms can be specifically targeted by pharmacological agents, an approach that was demonstrated to be effective in some tumours and neurological disorders and that also holds great promise for future treatment of bone diseases. In the setting of precision medicine, all these features can be exploited to grant tailored treatment for the individual.

## Figures and Tables

**Table 1:** Main epigenetic mechanisms and their principal effects on gene expression.

Epigenetic Process (Post-Translational Histone Modifications)	Molecular Mechanism	Involved Enzymes	Mechanism of Action	Effects on Gene Expression
Histone acetylation/deacetylation	The lysine residues at the N-terminal of histone tails are subjected to either addition (acetylation) or removal (deacetylation) of acetyl groups.	(1) Histone acetyltransferases (HATs);	Acetylation removes positive charges from lysine residues and reduces the affinity between histones and DNA, thereby opening the condensed chromatin structure, favouring the access to gene promoters.	Histone acetylation promotes gene expression. Conversely, histone deacetylation prevents gene expression.
		(2) Histone deacetylases (HDACs)		
Histone methylation/demethylation	Histone methylation occurs on different lysine residues, with the potential addition of one, two, or three methyl groups.	(1) Histone lysine methyltransferases (KMTs);	The effect of histone methylation on chromatin state is dependent not only on the specific lysine residue modified, but also on its degree of methylation.	Histone methylation at H3K4, H3K36, or H3K79 has been associated with gene transcription activation.
		(2) Histone lysine demethylases (KDMs)		Histone methylation at H3K9, H3K20, or H4K27 is implicated in gene expression inactivation or silencing.
DNA methylation	Addition of a methyl group at the 5′ position of the cytosine ring within CpG islands of gene promoters.	(1) DNA methyltransferases (DNMT3A and DNMT3B);	Methylated gene promoters are not accessible to transcription factors.	DNA methylation is strongly associated with gene transcription silencing.
		(2) DNA maintenance methyltransferase (DNMT1)		
MicroRNAs (miRNAs)	miRNAs selectively bind to the 3’ non coding region (3’UTR) of specific target mRNAs, through base-pairing.	None	Binding of a miRNA on the 3’UTR of the target mRNA blocks protein synthesis by two distinct post-transcriptional mechanisms: mRNA cleavage or translational repression.	miRNAs negatively regulate the expression of target genes, at post-transcriptional level, by blocking the translation of their proteins.

**Table 2:** Role of histone deacetylases (HDACs) in bone biology.

HDAC	Class	Affected Protein Expression	Effects on Bone Biology	Reference
HDAC1	I	RUNX2 (down-regulation)	Suppression of osteoblast differentiation	[6]
HDAC2	I	FoxO1 (down-regulation)	Promotion of RANKL-induced osteoclastogenesis	[13]
HDAC3	I	RUNX2 (down-regulation)	Maintenance of bone mass during development and aging	[3]
HDAC4	II	RUNX2 (down-regulation)	Suppression of endochondral ossification	[8,9]
HDAC5	II	RUNX2 (down-regulation)	Suppression of osteoblast differentiation	[8]
HDAC7	II	RUNX2 (down-regulation)	Regulation of endochondral ossification	[15]
HDAC8	I	Homeobox transcription factors Otx2 (up-regulation) and Lhx1 (up-regulation)	Regulation of intramembranous ossification	[11]
HDAC9	II	RANKL (down-regulation)	Suppression of osteoclastogenesis	[17]
Sirt1	III	NA	Promotion of endochondral ossification, and of osteoblast differentiation of mesenchymal stem cells	[17,18]
Sirt6	III	NA	Promotion of endochondral ossification	[17]

NA = non available.
